# Early noninvasive prenatal detection of a fetal *CRB1* mutation causing Leber congenital amaurosis

**Published:** 2008-08-04

**Authors:** Ana Bustamante-Aragones, Elena Vallespin, Marta Rodriguez de Alba, Maria Jose Trujillo-Tiebas, Cristina Gonzalez-Gonzalez, Dan Diego-Alvarez, Rosa Riveiro-Alvarez, Isabel Lorda-Sanchez, Carmen Ayuso, Carmen Ramos

**Affiliations:** Department of Genetics, Fundacion Jimenez Diaz-Capio, CIBERER, Madrid, Spain

## Abstract

**Purpose:**

Leber congenital amaurosis (LCA) is one of the most severe inherited retinal dystrophies with the earliest age of onset. Mutations in the *Crumbs homologue 1* (*CRB1*; OMIM 600105) gene explain 10%–24% of cases with LCA depending on the population. The aim of the present work was to study a fetal mutation associated to LCA in maternal plasma by a new methodology in the noninvasive prenatal diagnosis field: the denaturing High Performance Liquid Chromatography (dHPLC).

**Methods:**

This study presents the case of a compound heterozygous fetus for two mutations in *CRB1* (1q3.1-q32.2). dHPLC and automated DNA sequencing were used to detect the paternally inherited fetal mutation in a maternal plasma sample collected at the 12th week of gestation. To test the detection limit of dHPLC, we made serial dilutions of paternal DNA in control DNA.

**Results:**

We were able to detect the presence of the paternally inherited fetal *CRB1* mutation in maternal plasma by dHPLC. Moreover, by comparing chromatograms of serial dilutions to the plasma sample, we could ascertain that the percentage of fetal DNA in maternal plasma was at least 2%. However, the detection of the fetal mutation was not possible by automated DNA sequencing.

**Conclusions:**

dHPLC seems to be sensitive enough to detect small amounts of fetal DNA in maternal plasma samples. It could be a useful tool for the noninvasive prenatal detection of paternally inherited point mutations associated with retinopathies.

## Introduction

Leber congenital amaurosis (LCA; OMIM 204000) is a severe form of inherited retinal dystrophy with the earliest onset [1-6]. LCA is generally inherited in an autosomal recessive manner although some autosomal dominant families have been described [7-9]. Nonsyndromic LCA has so far been associated with mutations in 12 genes (RetNet).

Mutations in *Crumbs homologue 1* (*CRB1*; OMIM 600105) have been associated with several visual disorders including retinitis pigmentosa (RP) with [10,11] or without [11,12] preserved para-arteriolar retinal pigment epithelium, paravenous pigmented chorioretinal atrophy [13], and LCA [14,15]. Of these disorders, LCA is the most severe form of inherited retinal dystrophy and is characterized by severe visual impairment from birth or very early in infancy and a decreased or absent electroretinogram (ERG) response [16]. Mutations in *CRB1* explain 10%–24% of cases with LCA depending on the population [4-9,17-21].

The first evidence of the existence of fetal material in maternal tissue was discovered in 1893. Fetal cells were found in the lungs of pregnant women who died of eclampsia [22]. But it was in the 1980s when this discovery was taken into consideration, opening the possibility to develop a noninvasive prenatal diagnosis (NIPD) to avoid the risk that obstetric invasive techniques entail. Fetal sex assessment and the study of the most common aneuploidies were the first diagnoses carried out from the analysis of fetal cells in maternal blood [23,24]. In 1997, Lo et al. [25] discovered the existence of circulating cell free fetal DNA (ccffDNA) in maternal blood. This discovery was based on the detection of Y-chromosome specific sequences in the maternal circulation of pregnant women bearing a male fetus. Once the presence of this fetal DNA in maternal plasma was widely demonstrated [26-28], some studies focused on the quantification of this fetal material [29-31].These studies reported that the ccffDNA represented around 3%–6% of the total DNA present in the maternal plasma [29]. In addition, it was observed that the amount of ccffDNA increases throughout gestation [31] and disappears immediately after delivery [32].

An important limitation of these kinds of studies is that, because of the presence of maternal DNA in the plasma samples, they are bound solely to the detection of paternally inherited fetal sequences. Therefore, the ccffDNA present in maternal plasma has been mainly used for fetal gender assessment [26-28], determination of fetal rhesus status in Rh-negative pregnant women [33-35], and detection of paternally inherited disorders [36-41].

Because of the low percentage of ccffDNA present in maternal plasma, all studies require the use of sensitive technologies. Real-time PCR (RT-PCR) has been the most widely used technique for this aim because its high sensitivity enables the detection of small amounts of target DNA sequences [28,42-44]. However, other approaches, such as restriction analysis [37] or quantitative fluorescent PCR (QF-PCR) [45,46], have been also used. In addition to these techniques, our group described in a previous report the use of automated DNA sequencing for the detection of a paternally inherited fetal mutation associated with an X-linked RP [41].

Here, we present the first evidence of the application of denaturing high performance liquid chromatography (dHPLC) for the detection of an LCA-associated fetal mutation in maternal plasma. dHPLC is a technique currently used in diagnostic laboratories for the detection of point mutations or small deletions/insertions. However, to the best of our knowledge, this technology has not been applied in the NIPD field yet. The aim of this work is to report the use of this technique for the detection of a paternally inherited fetal mutation associated to LCA in maternal plasma in the first trimester of gestation.

## Methods

### Patients

**Figure 1:**
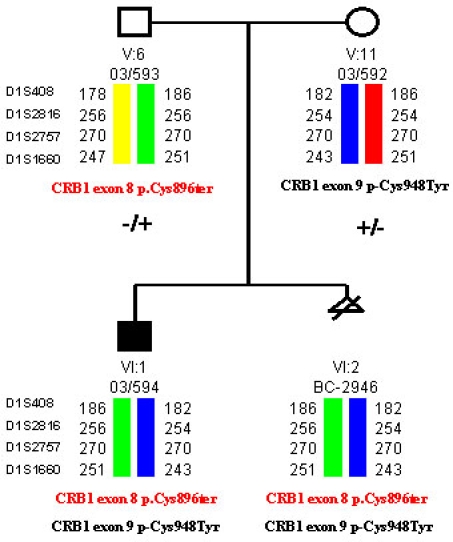
Genealogy of the family. Parents were carriers of a different mutation in *CRB1* (paternal mutation in red color). Haplotypes for four markers flanking the gene are showed (color bars). The first child (VI1) was affected with LCA. The probandus of the present study (VI:2) was carrier of both parental mutations.

In the present study, the patient was an in utero fetus. The parents of the fetus came to our clinic to undergo a chorion biopsy for prenatal diagnosis. The parents were heterozygous for two different mutations in *CRB1*: the father carried the p.Cys896ter mutation in exon 8 and the mother the p.Cys948Tyr mutation in exon 9 (Figure 1).

### Sample collection

Maternal blood (9 ml) was collected in EDTA tubes at the 12th week of gestation before the chorion biopsy was performed. This collection was done under informed consent and according to the Helsinki declaration. The sample was centrifuged at 1,600x g for 10 min. Plasma was collected in 1 ml fractions and centrifuged at 16,000x g for 10 min to eliminate all maternal cells present in the plasma sample. Supernatant was collected in new tubes and stored at –20 °C.

In the present study, chorion villi sample was used as fetal control, paternal DNA served as positive control for the p.Cys896ter mutation and maternal DNA was used as wild-type control for the p.Cys896ter mutation.

### DNA extraction

DNA was extracted from 2 ml of maternal plasma with the QIAmp DNA Blood MiniKit (Qiagen, Hilden, Germany) following the protocol recommended by the manufacturer with one modification: the eluate was reloaded into the column and centrifuged again. The parental DNAs were already available from previous studies. DNA extraction from the chorion villi sample was performed using the Tissue Extraction Kit in the BioRobot EZ1 (Qiagen) following the recommended protocol.

### Polymerase chain reaction amplification

*CRB1* is composed of 12 exons. The mutation carried by the father was in exon 8 (p.Cys896ter). A 276 bp fragment of exon 8 was amplified by PCR. Amplification was performed using 25 μl of DNA extracted from the plasma sample as a template. For controls (paternal DNA, maternal DNA, and chorion biopsy DNA), 2 ng of DNA were used as PCR control templates. The final volume of the reaction was 50 μl containing: 5 pmol of CRB1ex8 forward, 5′-CAA CAT TTT TCT ATT TAG TTG CC-3′ (Applied Biosystems, Foster City, CA); 5 pmol of CRB1ex8 reverse, 5′-CTC AAA TGT CGC AAC TTA ACT G-3′ (Applied Biosystems); 1X PCR buffer with 2.5 nM MgCl_2_ (Roche, Indianapolis, IN); 200 μM each deoxynucleotide; and 1U of FastStart Taq DNA Polymerase (Roche). Amplification was performed in a GeneAmp PCR System 2700 thermal cycler (Applied Biosystems). After an initial incubation of 95 °C for 10 min, the reaction was cycled for 30 s at 95 °C, 20 s at 60 °C, and 50 s at 74 °C for 40 cycles, followed by a final extension of 5 min at 74 °C. PCR products were analyzed by two different methods: dHPLC and automated sequencing.

### Denaturing high performance liquid chromatography

In this technique, the PCR products are denatured and subsequently subjected to a slow renaturalization. During renaturalization, complementary sequences perfectly reanneal, creating homoduplex forms. However, the presence of a different nucleotide in a specific position in one of the strands of DNA will generate mismatched double-stranded DNA fragments called heteroduplexes. dHPLC can reveal the presence of differences in the sequences based on the detection of the heteroduplex forms.

Denaturing of the PCR products was performed at 95 °C for 5 min, and posterior renaturing was done at room temperature for over 1 h. dHPLC analysis was performed using a WAVE^TM^ DNA fragment Analysis System (Transgenomic Inc., Omaha, NE). Subsequently, 5 μl of renatured products were loaded on a C18 reversed-phase column (DNASep^TM^ column; Transgenomic Inc.) and analyzed with an acetonitrile gradient formed by mixing buffers A and B (WAVE Optimized^TM^; Transgenomic Inc.). The optimal partial denaturing temperature used was 59.9 °C, which was calculated by the WAVE Maker program (Ver. 4.1; Transgenomic Inc.).

In order to know the detection limit of the dHPLC for the p.Cys896ter mutation, a battery of serial dilutions of paternal DNA (carrier control) in maternal DNA (wild-type DNA) was made. These dilutions were made with different ratios of paternal DNA/maternal DNA (% of paternal DNA): 50/50 (50%), 25/75 (25%), 15/85 (15%), 10/90 (10%), 5/95 (5%), 4/96 (4%), 3/97 (3%), 2/98 (2%), and 1/99 (1%).

### Automated sequencing

PCR products were purified by QIAGEN Purification Kit columns (Qiagen). The plasma sample was eluted in 30 µl of elution buffer and the controls in 50 µl. Based on the manufacturer’s recommendations, the sequencing reaction was performed in a final volume of 20 μl containing 5 μl of purified PCR product for the controls and 10 μl for the plasma sample, 10 pmol of CRB1ex8 forward or reverse primer, and dRhodamine terminator cycle sequencing ready reaction kit (Applied Bioscience). The product was electrophoresed in an ABI Prism 3100 Genetic Analyzer (Applied Bioscience) and analyzed with the Sequencing Analysis 5.1.1 software package (Applied Bioscience).

## Results

### Denaturing high performance liquid chromatography analysis

**Figure 2:**
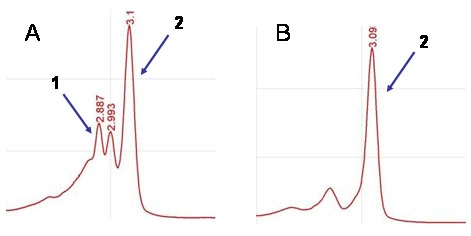
Chromatograms of the control DNAs obtained by dHPLC. After PCR amplification of exon 8 of *CRB1* (where the p.Cys896ter mutation is located), the PCR products were analyzed by dHPLC. **A:** Electropherogram showed by the paternal DNA (carrier for the p.Cys896ter mutation). 1: Double-peak generated by the heteroduplexes forms associated to the presence of the p.Cys896ter mutation in the sample. 2: Peak generated by the homoduplexes. The presence of both homo and heteroduplexes peaks indicates a heterozygous genotype for the p.Cys896ter mutation. **B:** Electropherogram showed by the maternal DNA (wild-type for the p.Cys896ter mutation). 2: Peak generated by the homoduplexes. The only presence of homoduplexes peak represents a wild-type genotype.

dHPLC analysis of the control DNA for the mutation in exon 8 (maternal DNA) and the heterozygous mutant control (paternal DNA) revealed two different and distinguishable chromatograms. The control DNA chromatogram showed a unique peak generated by the homoduplex forms. However, the heterozygous mutant control presented a different pattern comprised three peaks: one peak corresponding to the homoduplexes, and two smaller additional peaks corresponding to the heteroduplex forms (Figure 2).

**Figure 3:**
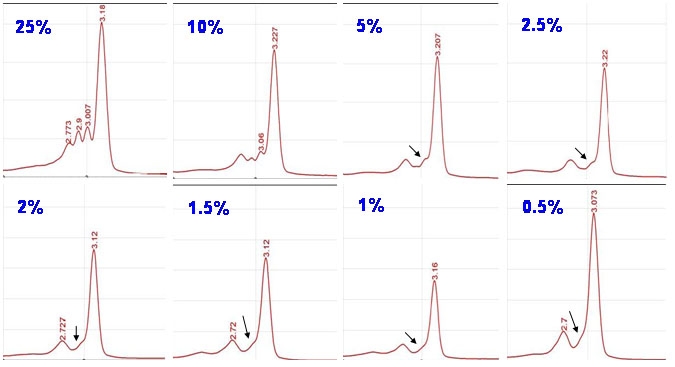
Chromatograms showed by the analysis of the serial dilutions by the dHPLC. The percentage data indicate the amount of the carrier DNA present in the dilution. The double-peak corresponding to the heteroduplexes decreases in size as the percentage of mutant DNA in the sample gets lower. From the 5% to the 2% dilution, the double-peak becomes a slight peak (pointed by the arrows in the figure). Below the 2%, the heteroduplexes could not be distinguished. These chromatograms revealed that the mutation was detectable up to the 2% dilution.

Chromatograms from the serial dilutions showed how the two peaks corresponding to the heteroduplex forms diminished as the amount of paternal DNA in the mix decreased. In mixes with low concentrations of paternal DNA, these two peaks became a “slight peak” which was visible until the 2/98 (2%) dilution. When the mutation was in a percentage lower than 2% it was not detectable because the chromatogram was similar to the one showed by the maternal DNA. (Figure 3)

**Figure 4:**
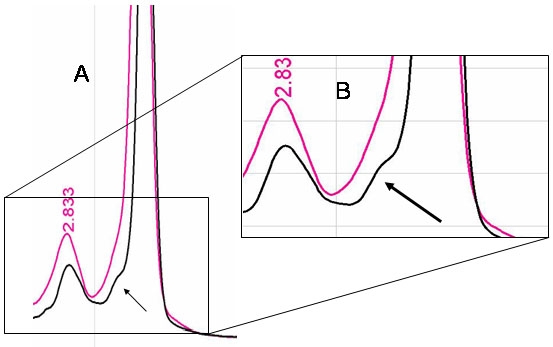
Analysis of the maternal plasma sample by dHPLC. **A:** Chromatograms from the maternal plasma sample (black graph) and the wild-type control DNA chromatogram (pink graph). They are shown overlapped in order to be compared. The arrow points the peak shape in the graph associated to the presence of the p.Cys896ter mutation in the maternal plasma sample and not present in the wild-type chromatogram; **B:** Enlarge image from the region of interest revealing the carrier condition of the fetus for the p.Cys896ter mutation.

Analysis of the plasma sample showed a chromatogram in which the ‘slight peak’, previously observed in the 5% and 2% dilution controls and associated to the mutation, was present (Figure 4). The presence of this slight peak in the maternal plasma sample chromatogram represented the presence of the paternally inherited fetal mutation.

The analysis of the chorion biopsy revealed that the fetus had inherited the paternal mutation.

### Sequencing analysis

The detection of the paternal mutation (p.Cys896ter) was determined by the presence of an adenine (instead of a thymine) at the mutation site (c.2688 T>A). We were not able to detect the paternal mutation in the plasma sample by automated sequencing. However, the mutation was observed in the analysis of the chorion villi sample.

## Discussion

Different techniques have been applied to detect fetal DNA in maternal plasma. The tools most commonly used are RT–PCR, QF-PCR, and restriction analysis. However, the adoption of new emerging techniques, usually more sensitive, may increase the possible diagnoses to be offered by the noninvasive prenatal diagnosis.

In the present study, the parents of the fetus were heterozygous for a different mutation in different exons of *CRB1*.

The aim of this work was to introduce the dHPLC technology for the detection of a paternally inherited fetal mutation associated to LCA in maternal plasma in the first trimester of gestation. Exclusion of the paternal mutation in the maternal plasma would indicate that the fetus was, at worst, a carrier of the maternal mutation. Therefore, conventional invasive procedures could be avoided.

Used as a screening tool, the dHPLC technique searches for unknown mutations in large scale studies or known mutations in new patients with a previously diagnosed relative. Some advantages of this tool are its easy handling, its ability to rapidly process samples, and its low cost. Moreover, it has shown excellent results in clinical practice [47].

Considering the low percentage of ccffDNA in the maternal plasma previously reported [29] and in order to know the detection limit of the dHPLC for the p.Cys896ter mutation, serial dilutions of paternal DNA (carrier for the mutation) were made. In addition, the comparison of the migration patterns between the serial dilutions and the plasma sample was the strategy followed to determine the genotype of the fetus for the paternal mutation.

Analysis of the chromatograms from the dilutions helped to establish the detection limit of the technique for the p.Cys896ter mutation as 2% of carrier DNA present in the sample. This high level of detection made us look at this technique as a promising tool for the detection of fetal DNA in maternal blood. This hypothesis was confirmed when we were able to detect the mutation by analyzing the maternal plasma sample.

The correspondence of the migration patterns of the serial dilutions (5% and 2% dilutions) with the one showed by the plasma sample let us ascertain the carrier condition of the fetus for the paternal mutation. Besides, this assay design also allowed us to speculate that the amount of fetal DNA represented around 2% to 5% of the total DNA present in the plasma sample analyzed.

In a previous paper, we reported the detection, by automated sequencing, of a fetal mutation associated to a X-linked Retinitis Pigmentosa in maternal plasma collected at the 19th week of gestation [41]. However, a sample collected from the same pregnancy at the 10th week was also analyzed but the mutation was not detected [41]. In the present study, the paternally inherited fetal mutation has been detected in the maternal plasma (at the 12th week) by the dHPLC method but not by automated sequencing. Therefore both studies, in which automated sequencing has been used, are concordant about the inefficiency of the technique for detection of ccffDNA in maternal plasma samples in the first trimester of gestation. This fact would be in accordance with previous reports about the scarcity of ccffDNA in the first trimester of gestation [30,31].

This work opens up the possibility to incorporate the dHPLC technique for the study of paternally inherited fetal mutations in maternal plasma. It has shown to be sensitive enough to detect ccffDNA in the first trimester of gestation. However the automated sequencing technique has not been efficient for the detection of ccffDNA at this early stage of gestation. The creation of serial dilutions containing low percentages of paternal DNA has shown to be an essential strategy for the analysis of paternally inherited fetal mutations in maternal plasma by dHPLC. Considering that the detection limit of the technique could be variable for the analysis of different mutations, further studies are required to evaluate the accuracy of the method for other mutational changes.
